# Analysing the influence of foreign direct investment and urbanization on the development of private financial system and its ecological footprint

**DOI:** 10.1007/s11356-022-22772-9

**Published:** 2022-09-03

**Authors:** Pablo Ponce, José Álvarez-García, Viviana Álvarez, Muhammad Irfan

**Affiliations:** 1 Carrera de Economía y Centro de Investigaciones Sociales y Económicas, Universidad Nacional de Loja, 1050 Loja, Ecuador; 2 Faculty of Economics and Business, University of Vigo, Campus Universitario, s/n, 36310 Vigo, Spain; 3 Departamento de Economía Financiera y Contabilidad, Instituto Universitario de Investigación para el Desarrollo Territorial Sostenible (INTERRA), Universidad de Extremadura, 10071 Caceres, Spain; 4 School of Management and Economics, Beijing Institute of Technology, Beijing, 100081 China; 5 Center for Energy and Environmental Policy Research, Beijing Institute of Technology, Beijing, 100081 China; 6 Department of Business Administration, ILMA University, Karachi, 75190 Pakistan

**Keywords:** Financial development, Ecological footprint, Foreign investment, Urbanization, Panel data estimation

## Abstract

In this research, the objective is to examine how private financial development, urbanization and foreign direct investment and economic growth affects the environment using the ecological footprint as an indicator. Panel data was used for 100 countries from 1980 to 2019, classified according to their income level. Several econometric steps were used to estimate the results, such as cointegration and causality techniques. The results show that the private financial system and environmental degradation have a long-term equilibrium relationship, and the incidence is positive, but not significant at the level of the 100 countries. In high-income countries, the private financial system reduces environmental degradation; however, in upper middle-income, lower middle-income and low-income countries, it increases in the long run. Likewise, urbanization plays a predominant role on the ecological footprint in the long term. Meanwhile, the role of foreign direct investment is not stable over time. The causality test shows bidirectional causality between environmental degradation and the private financial system at the global level in high- and upper middle-income countries. However, low-income countries have a unidirectional relationship of environmental degradation to the private financial system. With regard to foreign direct investment, there is a unidirectional causal relationship between environmental degradation and foreign direct investment at the global level and from foreign direct investment to environmental degradation in high-income countries.

## Introduction

Environmental degradation is an issue of constant concern, given that it involves climate change, loss of ecosystems and global warming (Shujah-ur-Rahman et al. 2019a; Saleem et al. 2019). Since the 1950s, it has increased significantly, representing a threat and a challenge to achieving sustainable development (Ulucak et al. 2019). Currently, more than 80% of the world’s population is located in countries with ecological deficit, due to the fact that the population exceeded the Earth’s biocapacity, which in 2019 was 1.6 global hectares (gha) per person, and in that same year, 2.77 gha per person was required to provide the natural resources consumed by the population (GFN 2021). Furthermore, the World Bank (2019) revealed that 60–70% of the world’s ecosystems are deteriorating at an accelerated rate, showing that a change in the current development model is required.

Furthermore, the United Nations Environment Programme (UNEP 2021) indicated that since 2010, global greenhouse gas emissions have increased by 1.4% on average per year, and in 2019, they increased to 2.6%, due to the increase in forest fires (Huang et al. 2022). Similarly, global carbon dioxide (CO_2_) emissions increased by 14.1% during the period 2009–2018. However, by 2020, they had decreased by 5.8%, due to the COVID-19 pandemic that reduced the demand of the industry, transport and electricity and heat producing sectors, which are the largest CO_2_ emitters. Nevertheless, by 2021, it is predicted that with the global economic recovery, the demand for resources will be at pre-pandemic levels, or even higher, and CO_2_ emissions will increase along the same lines (IEA 2021).

According to the International Energy Agency (IEA 2021), developing economies currently make up more than two-thirds of global CO_2_ emissions, while emissions from developed economies have declined, accounting for less than one-third of global CO_2_ emissions. For high-income countries (HIC), CO_2_ emissions per person decreased by 9.9% during the period 2010–2018; in the case of upper middle-income countries (UMIC), emissions increased by 2.71% in the period 2016–2018; for lower middle-income countries (LMIC), emissions increased by 41.04% during the period 1999–2018; and finally, for low-income countries (LIC), emissions decreased by 8.33% during the period 2016–2018 (World Bank 2019).

Different investigations have focused on studying the relationship between financial development and environmental degradation (Irfan et al. 2022), which is a controversial topic due to its sign and magnitude (Ibrahiem 2020; Bui 2020; Avom et al. 2020; Shujah-ur-Rahman et al. 2019b). On the one hand, it was established that the financial sector has a positive and significant effect on long-term environmental degradation, by enhancing industrial activities (Ibrahiem 2020; Avom et al. 2020; Jiao et al. 2021). Meanwhile, other researchers argue that private financing is crucial to reduce environmental degradation by financing sustainable and efficient projects (Salahuddin et al. 2020; Dogan et al. 2019). In addition, variables such as foreign direct investment (*FDI*), urbanization and economic growth were included, which can affect the ecological footprint positively or negatively in the long term depending on countries’ income and the rigour of their environmental policies (Doytch 2020; Danish and Wang 2019; Adams and Nsiah 2019).

On the other hand, it is also important to mention that in the current empirical literature, researchers generally use CO_2_ emissions as a proxy measure of environmental degradation. However, there are limitations, since complete information on the environmental damage caused is not revealed (Ahmed et al. 2021). In this regard, recent studies used the ecological footprint (*EF*) as a more comprehensive measure of environmental degradation, as it includes six productive areas: agricultural land, livestock land, fisheries sectors, forestry land, infrastructure land and carbon footprint (Omoke et al. 2020; Sharma et al. 2021c; GFN 2021). However, this document contributes to fill the gap in the current literature, in relation to the ecological footprint, with countries grouped with different income levels. Previous studies have focused on performing an ecological footprint analysis (Sharma et al. 2022, 2019; Sharma et al. 2021a); however, according to the exhaustive review of the literature, there are few that focus on a global level.

In this context, the aim of this research is to evaluate the causal relationship between the private financial system and environmental degradation. The research covers the period 1980–2019, for 100 countries grouped by income. Cointegration techniques, ARDL dynamic models, and the Granger causality test (1969) developed by Dumitrescu and Hurlin (2012) were used. This research contributes to the scientific field in various ways:

1. It helps to understand how the development of the private financial system affects the degradation of the environment, in various countries with different income levels. Likewise, it uses other exogenous factors, such as foreign direct investment and urbanization. Both cited in the literature as drivers of environmental degradation.
2. It uses the ecological footprint as a measure of environmental degradation, which is a more accurate measure than the conventional ones.
3. The results found are extremely solid, given that it uses second-order econometric techniques, that is, the results of the environmental degradation analysis are unbiased, since the cross-section dependency is controlled.
4. The panel econometric estimators used are mean group (MG) and pooled mean group (PMG). These estimators capture the performance of environmental degradation considering the behaviour of this variable in previous periods. Likewise, it allows examining the degradation of the environment in the short and long term. Consequently, the contributions of the study provide important findings to public policy decision-makers to establish mechanisms to mitigate environmental degradation, enabling them to develop strategies and economic measures for a sustainable environment.

The document is structured in 5 sections. The “Introduction” contextualises the topic and sets out the aim of the research, as well as the novelty of the research. The second section contains a review of the relevant literature on the subject of study. The third section describes the data used and the econometric strategy. In the fourth section, the results are discussed and contrasted with the existing empirical evidence. The fifth and final section presents the findings and policy implications of the research.

## Previous literature review

Environmental degradation has been an issue of constant concern in recent years, given that humanity has surpassed the planet’s biocapacity. In this regard, several academics have focused on studying the impact of different variables on environmental degradation. Empirical evidence will be grouped into four groups. The first group includes studies that relate the private financial system to environmental degradation, and the second group includes studies that examine the effect of foreign direct investment on environmental degradation. The third group includes studies linking urbanization and environmental degradation. Finally, the fourth group includes studies that explain the relationship between economic growth and environmental degradation.

### Private financial system and environmental degradation

The financial system plays a key role in the savings and investment process by facilitating the circulation of monetary resources in the economy. However, it must take environmental and social factors into account in order to be sustainable (Xie et al. 2022; Ziolo et al. 2019). In the studies carried out in relation to the environmental degradation and the private financial system, most researchers used as a measure of environmental degradation the carbon dioxide (CO_2_) emissions (Khan et al. 2019; Fang et al. 2020). Nevertheless, recent research indicates that the *EF* is a comprehensive measure for determining environmental degradation (Omoke et al. 2020; Sharma et al. 2021c).

There are studies within this group which show that the private financial system contributes to environmental degradation and that actions are required to mitigate its impact (Wang et al. 2020a, b). Research using CO_2_ emissions corroborated that private credits increase environmental degradation, due to increased investment and consumption in polluting activities (Ibrahiem 2020; Bui 2020; Avom et al. 2020). In the same way, research incorporating the ecological footprint as a measure of environmental degradation established that the impact of financial resources is positive on the *EF* in the long term, due to the establishment and expansion of businesses, increased purchasing power of the population and infrastructure projects, which lead to increased environmental pressure (Baloch et al. 2019; Godil et al. 2020; Ahmed et al. 2021). They also demonstrated that there is a bidirectional relationship between the variables (Saud et al. 2020; Shujah-ur-Rahman et al. 2019c).

In contrast, other studies show that the private financial system leads to an improvement in the quality of the environment, as it decreases CO_2_ emissions (Haider-Zaidi et al. 2019; Villanthenkodath and Arakkal 2020; Salahuddin et al. 2020; Aluko and Obalade 2020). Likewise, Dogan et al. (2019), Destek and Sarkodie (2019), Usman et al. (2020), Usman and Hammar (2021) Queryand Sharma et al. (2021c) established that private credits have a negative effect on the long-term ecological footprint, indicating that financial assets are used efficiently for environmental improvement. Furthermore, Dogan et al. (2019) and Destek and Sarkodie (2019) reported the existence of a unidirectional causality from the ecological footprint to the financial system and from the financial system to the ecological footprint, respectively. On the other hand, Usman et al. (2020) and Usman and Hammar (2021) found bidirectional causality between the variables. On the other hand, other researchers, such as Charfeddine and Kahia (2019) and Abokyi et al. (2019), established that the contribution of financial development to environmental quality is not significant for some countries.

### Foreign investment and environmental degradation

The flow of foreign direct investment (*FDI*) can have positive or negative effects on the host country’s economy and environment (Yilanci et al. 2020). Rafindadi et al. (2018) stated that higher *FDI* increases CO_2_ emissions in the short term. However, in the long term, the reverse effect will occur. On the other hand, Hanif et al. (2019), Zafar et al. (2020) and Xie et al. (2020) state that the contribution of *FDI* is significant and positive on CO_2_ emissions in the long term. In order to have a higher economic growth, they soften their environmental standards, especially in developing countries, becoming a profitable and low-cost environmental target for highly polluting companies (Opoku and Boachie 2020; Essandoh et al. 2020; Sarkodie and Strezov 2019). In the same vein, studies using the ecological footprint as a measure of environmental degradation are presented. Doytch (2020), Murshed et al. (2021) and Sabir et al. (2020) indicated that *FDI* has a negative impact on environmental quality, especially in developing countries. Furthermore, the bidirectional relationship between the variables is shown (Sabir et al. 2020).

On the contrary, there are studies that claim that *FDI* is positive for improving environmental quality because foreign companies have advanced and efficient technologies that contribute to reducing CO_2_ emissions (Hille et al. 2019; Eluwole et al. 2020; Opoku et al. 2021). Similarly, the study by Zafar et al. (2019) used the ecological footprint as a measure of environmental degradation and it was argued that *FDI* shows a negative and significant impact on the ecological footprint in the long term, in addition to the existence of bidirectional causality between the variables. Finally, the study by Mahmood et al. (2020) pointed out that the effect of *FDI* on environmental quality is statistically insignificant. Therefore, the type of impact that *FDI* will have on environmental degradation cannot be examined.

### Urbanization and environmental degradation

The urbanization process observed in different countries can have different impacts depending on the economies studied. Asongu et al. (2020) and Ridzuan et al. (2020) investigated the relationship between urbanization and environmental degradation, finding that an increase in urbanization will have a positive effect on CO_2_ emissions in the long term. However, Ridzuan et al. (2020) claim that in the short term, its impact will be negative. In contrast, other authors using the ecological footprint as a measure of environmental degradation found that the effect of urbanization on the ecological footprint is positive in the short and long term (Ahmed et al. 2020; Langnel and Amegavi 2020). According to Ahmed et al. (2020), Ulucak et al. (2020) and Langnel and Amegavi (2020), this is due to the fact that urbanization affects economic and social activities, which demand greater energy consumption in households and production sectors. In addition, they do not have planned urban systems, resulting in increased environmental pressures (Liang et al. 2022; Sun et al. 2022).

On the other hand, other researchers found that urbanization contributes to mitigating environmental degradation. This will occur if variables such as economic growth play a moderating role, since, with economic growth, urbanization will reach a level of development at which environmental damage will begin to be reversed, showing positive externalities and greater environmental awareness among the population (Danish and Wang 2019). The study by Dogan et al. (2019) found that the impact of urbanization is negative on the ecological footprint in the long term for Nigeria, showing the difference in the urban planning techniques applied, in addition to the existence of a unidirectional relationship between urbanization and the ecological footprint. Finally, in the study by Arshad Ansari et al. (2020) for Asian regions, it was found that urbanization is not statistically significant on the ecological footprint.

### Economic growth and environmental degradation

The relationship between economic growth and environmental degradation was analysed by several researchers, establishing that economic growth contributes to further environmental degradation (Adams and Nsiah 2019; Nathaniel and Adeleye 2021; Sharma et al. 2021b). Along the same lines, Wang et al. (2020a, b) and Danish Ulucak and Khan (2020) indicated the existence of an inverted U-shaped relationship between GDP and the ecological footprint, arguing that in the early stages of development, environmental degradation increases because no attention is paid to the environmental issue. However, from a specific level of income, the situation is reversed. The existence of unidirectional causality of GDP to the ecological footprint was also revealed, at a significance level of 5%. Similarly, Sharif et al. (2020) and Ahmad et al. (2020) concluded that economic growth and the ecological footprint have a positive long-term relationship, in addition to corroborating the fulfilment of the EKC and determining the existence of bidirectional causality between the variables.

In contrast, Ulucak et al. (2020) observed a growing relationship between economic growth and the ecological footprint, indicating that these countries have not yet reached the level of per capita income to begin to reverse the unfavourable environmental situation they face. Similarly, Destek and Sinha (2020) observed in their study that as income increases, less environmental degradation will be experienced. However, after reaching a particular level of economic growth, the opposite will occur. Likewise, Sharma et al. (2021a, b, c, d) reveal that economic growth is an instrument to mitigate environmental degradation in Asian economies. On the other hand, Uddin et al. (2019) and Nathaniel and Khan (2020) found that economic growth contributes to environmental degradation in the long term and inferred that if the countries under study and the world in general continue to pursue accelerated economic growth without taking the environment into account, there will be environmental damage that cannot be reversed. Additionally, these findings are corroborated by Sharma et al. (2021b), who indicate that economic growth drives environmental degradation.

## Data and methodology

### Data

This research evaluates the causal effect of the private financial system on environmental degradation. This research is applied to 100 countries in the world; the sample is limited by the availability of data for certain countries. Data from the period 1980–2019 were used. The variable used to measure environmental degradation (dependent variable) is the ecological footprint per capita, which is measured in global hectares per capita. This variable is a comprehensive measure to determine the effect that human actions have on the environment (Al-Mulali and Ozturk 2015; Ahmed et al. 2021). Data were collected from the Global Ecological Footprint Network (GFN 2021), available until 2017, and they were extrapolated up to 2019. An autoregressive integrated moving average (ARIMA) model was used for data extrapolation. For which, each of the variables of each country was treated as time series to find the values of the years 2018 and 2019, respectively.

On the other hand, the independent variable is the private financial system, measured by domestic credits to the private sector as a percentage of GDP. This variable provides information on the volume of funds channeled to the private sector by financial institutions. In addition, foreign direct investment, urbanization and gross domestic product were considered control variables, which provide robustness to the model and are used as determinants of environmental degradation in studies developed by Nathaniel and Khan (2020) and Zafar et al. (2020). Data on the above variables are obtained from the World Development Indicators (World Bank 2021a, b). The description of the study variables is shown in Table 1.

**Table 1:** Description of variables used

Variable type	Variable and notation	Unit of measurement	Data source	Definition
Dependent	Ecological footprint (${EF}_{pc}$)	Global hectares	GFN	It measures human supply and demand for the planet’s ecological resources per person
Independent	Private financial system ($$PFS)$$	Percentage of GDP	World Bank	Domestic credit to the private sector; they are the financial resources provided to the private sector by financial corporations
Control	Foreign direct investment $(FDI)$	Percentage of GDP	World Bank	Capital investment made by an individual or entity in a country other than that of the investor
	Urbanization $(URB)$	Percentage of total population	World Bank	It refers to people living in urban areas
	Gross domestic product $({GDP}_{pc})$	Dollars	World Bank	Economic value of final goods and services produced in a country, in a given period, divided by population

Furthermore, the countries that make up the study sample are classified according to the World Bank’s Atlas method (Table 2). This classification is made considering the income levels that the countries have, in order to be able to examine the existing heterogeneity that they have according to each group of countries. This methodology classifies countries according to their income level into four groups: high-income countries (HIC) if their income is over USD 12,375, upper middle-income countries (UMIC) if their income is between USD 3996 and USD 12,375, lower middle-income countries (LMIC) if their income is between 1026 and 3995 and low-income countries (LIC) if their income is 1025 and less. In this way, a more focused and specific analysis can be carried out.

**Table 2:** Classification of countries according to their income level

Atlas classification	Countries
High-income countries (HIC)	Antigua and Barbuda, Barbados, Australia, Austria, Bahamas, Bahrain, Belgium, Chile, Cyprus, Denmark, Finland, France, Germany, Greece, Ireland, Israel, Italy, Japan, Rep. From Korea, Malta, Mauritius, Netherlands, New Zealand, Norway, Panama, Portugal, Spain, Sweden, Trinidad and Tobago, United Arab Emirates, the UK, the USA
Upper middle-income countries (UMIC)	Argentina, Botswana, Brazil, China, Colombia, Costa Rica, Dominica, Dominican Republic, Ecuador, Fiji, Gabon, Grenada, Guatemala, Guyana, Indonesia, Iran, Islamic Republic, Jamaica, Jordan, Malaysia, Mexico, Paraguay, Peru, South Africa, Saint Lucia, Thailand, Turkey
Lower middle-income countries (LMIC)	Algeria, Bangladesh, Benin, Bolivia, Cameroon, Ivory Coast, Egypt, El Salvador, Eswatini, Ghana, Honduras, India, Kenya, Lesotho, Mauritania, Morocco, Nicaragua, Nigeria, Pakistan, Papua New Guinea, Philippines, Rep. Congo, Senegal, Sri Lanka, Tunisia, Zambia, Zimbabwe
Low-income countries (LIC)	Burkina Faso, Burundi, Central African Republic, Chad, Rep. Dem. Congo, Gambia, Haiti, Madagascar, Malawi, Mali, Niger, Rwanda, Sierra Leone, Sudan, Togo

Table 3 shows the descriptive statistics of the study variables. It is observed that the ecological footprint has a greater variability between countries than within them; the standard deviation between countries is 1.94 and within countries it is 0.82. In addition, in general it has a minimum of 0.01 and a maximum of 9.99. Similarly, the private financial system is more stable within countries than between them. This can be observed in the standard deviation values, which is 20.74 within and 36.04 between. These data reflect that some countries have a better financial system than others.

**Table 3:** Descriptive statistics

Variable		Mean	*SD*	Min	Max	Remarks
${EF}_{pc}$	General	2.7791	2.0942	0.0109	9.9853	$N =4000$
	Between		1.9378	0.0212	8.0374	$n = 100$
	Inside		0.8168	− 3.5410	8.6526	$T = 40$
$PFS$	General	47.0167	41.4271	0.4914	253.262	$N =4000$
	Between		36.0392	2.7072	173.028	$n = 100$
	Inside		20.7377	− 40.6919	157.639	$T = 40$
$FDI$	General	3.5896	14.7398	− 39.5459	449.0828	$N =4000$
	Between		6.3176	0.1585	48.5615	$n = 100$
	Inside		13.3319	− 61.7979	404.1109	$T = 40$
$URB$	General	52.9625	23.1996	4.339	98.041	$N =4000$
	Between		22.6049	8.3802	96.9559	$n = 100$
	Inside		5.6762	20.0918	75.8250	$T = 40$
${GDP}_{pc}$	General	11,842.51	16,477.73	208.07	116,232.8	$N =4000$
	Between		15,932.17	260.26	75,188.91	$n = 100$
	Inside		4323.75	− 14,841.9	68,232.78	$T = 40$

*SD*, standard deviation; *N*, number of observations; *n*, number of panels; *T*, average number of years under observation

On the other hand, control variables such as urbanization and GDP per capita show a similar behaviour to the previous variables, since greater variability can be observed between countries than within each one. However, foreign direct investment (*FDI*) shows a different behaviour, having greater variability within countries (13.33) than between countries (6.32). In other words, each of the countries has a higher *FDI* in some years compared to other years of the study period, in which it is lower, reaching a minimum of − 39.55.

**Fig. 1:**
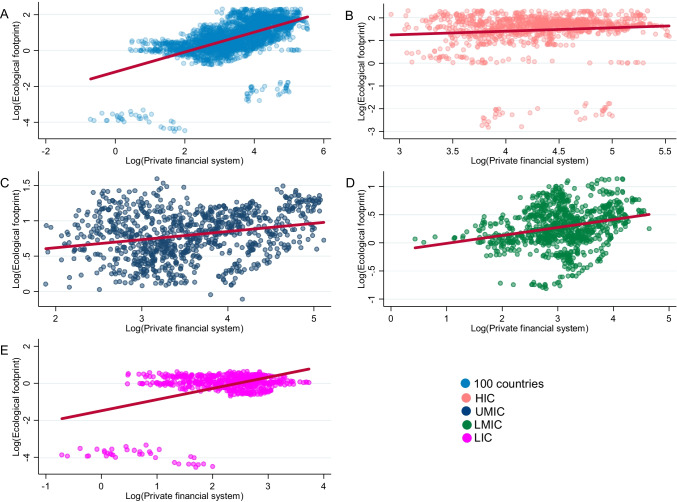
Correlation between the private financial system and environmental degradation (1980–2019)

Figure 1 shows the correlation between the private financial system and environmental degradation. In panel A, B, C, D and E, it can be seen that the correlation is significant and direct between domestic credits to the private sector and the ecological footprint at the global, HIC, UMIC, LMIC and LIC levels, respectively. In other words, as the private financial system develops, so does the ecological footprint. This situation shows that countries with greater access to private financial resources play an important role on the ecological footprint, since credits are used to finance activities that exert greater pressure on natural resources.

### Methodology

The cointegration and causality methodology, used in similar studies conducted by Nasir et al. (2019) and Fang et al., (2020), was applied. First, the function on which this research will be based according to the proposed variables will be described in detail. We will start from a baseline regression, where the ecological footprint (*EF*) is a function of the private financial system (*PFS*), which is shown in Eq. (1), considering that panel data are used.

1 $$\mathrm{log}({EF}_{i,t}) = ({(\beta }_{0} + \delta 1)) +{\beta }_{1}\mathrm{log}({PFS}_{i,t})+ {\mu }_{i,t}$$

where $\mathrm{log}({EF}_{i,t})$ represents the logarithm of the ecological footprint and $\mathrm{log}({PFS}_{i,t})$ represents the logarithm of private financial system of country *i* = 1, …,100, during *t* = 1980, …, 2019. On the other hand, the parameter ${\beta }_{0}$ collects the temporal variability; in other words, it determines how the constant varies over time. Likewise, the parameter ${\beta }_{1}$ captures the variability in the cross sections, that is, it will allow establishing how the constant varies for each country in the panel, while the parameter ${\mu }_{i,t}$ represents the stochastic error. In addition, the influence of control variables such as foreign direct investment (*FDI*)*,* urbanization (*URB*)and gross domestic product per capita (*GDPpc*) which will be included in the model is examined to provide robustness and obtain better results. In this regard, Eq. (2) shows the new model, including the control variables.

2 $$\mathrm{log}({EF}_{i,t}) = ({(\beta }_{0} + \delta 1)) +{\beta }_{1}{\mathrm{log}(PFS}_{i,t})+{\beta }_{2}{Z}_{i,t}+ {\mu }_{i,t}$$

where ${Z}_{i,t}$ is a vector that represents all the control variables such as: foreign investment, urbanization and economic growth. Considering what is intended to be analysed and in order to respond to the stated objective, autoregressive distributed lag (ARDL) techniques are estimated for panel data. This represents an advantage, since they allow for greater heterogeneity between parameters. Therefore, the econometric process is divided into four parts:

#### Cross-sectional dependence test

Initially, it is necessary to identify the presence of cross-sectional dependence (CD) in the data, since it is a common problem when using panel data. Omitting this problem causes misleading and biased results (Baltagi et al. 2016). Moreover, it is a key test to determine which unit root tests are appropriate to use in the research. Thus, the CD test developed by Pesaran (2004) is used, which is shown in Eq. (3).

3 $$CD=\sqrt{\frac{2}{N(N-1)}}\sum_{i=1}^{N-1}\sum_{j=i+1}^{N}{T}_{ij}{\widehat{p}}_{ij}^{2}$$

where $N$ represents the dimension of the cross section, $T$ is the period and ${\widehat{p}}_{ij}^{2}$ represents the correlation between the *i* and *j* residual coefficient.

#### Unit root test

The study variables may have non-stationarity problems, and spurious regressions may occur. Therefore, unit root tests are applied to indicate the order of integration of the variables. In this research, given the existence of cross-sectional dependence (CD) in the sections, it is appropriate to perform second-generation tests. In this regard, the cross-sectional augmented Im, Pesaran and Shin (CIPS) test suggested by Pesaran (2007) is used. It is suitable to control for slope heterogeneity and cross-sectional dependence. This test is shown in Eq. (4).

4 $$CIPS=\frac{1}{N}\sum_{i=1}^{N}{CADF}_{i}$$

where $CIPS$ represents the cross-sectional augmented Im, Pesaran and Shin test, $N$ represents the number of panels and ${CADF}_{i}$ represents the cross-sectional augmented Dickey-Fuller test, which is broken down into Eq. (5).

5 $$\Delta {x}_{it}= {\alpha }_{it}+{\beta }_{i}{x}_{it-1}+ {\rho }_{i}T+\sum_{j=0}^{n}{\theta }_{it}\Delta {x}_{it-j}+{\varepsilon }_{it}$$

where $\Delta {x}_{it}$ represents the variables analysed, ${\varepsilon }_{it}$ represents the residuals of the model, $i$ represents the cross-sectional dependence in the panel and $t$ represents the period.

#### Panel cointegration test

At this stage, the Westerlund (2007) cointegration test is performed, which makes it possible to establish if the set composed of variables is co-integrated in the long term. This test is made up of four cointegration statistics, which divides them into two groups, the first pair being the statistics of group *G*_*t*_ and *G*_*a*_, where the null hypothesis is that there is no panel cointegration and as an alternative that there is cointegration for the whole group. On the other hand, the second pair are the panel statistics *P*_*t*_ and *P*_*a*_, which indicate as an alternative hypothesis that there is cointegration in at least one cross-sectional unit against the null hypothesis that there is no cointegration. In addition, the *bootstrap* approach is applied, which allows to obtain more robust critical values. Equation (6) shows Westerlund’s (2007) cointegration:

6 $${y}_{i,t}={\delta }_{i}{d}_{t}+{\alpha }_{i}\left({y}_{i,t-1}-{\beta }_{i}{X}_{i,t-1}\right)+\sum_{j=1}^{{p}_{i}}{\alpha }_{ij}{y}_{i,t-j}+\sum_{j= {-q}_{i}}^{{p}_{i}}{y}_{ij}{X}_{i,t-j}+{\varepsilon }_{i,t}$$

where *t* = 1, …, *T* and *i* = 1, …, *N*, *d*_*t*_ denotes the deterministic components, while *p*_*i*_ and *q*_*i*_ are the lag and lead orders, which may vary in each country.

#### Dynamic panel techniques

After the previous tests, three different estimators of the ARDL model are used in this study to determine the short- and long-term equilibrium between the variables. The dynamic models used include the following: the mean group (MG) model of Pesaran and Smith (1995) and the pooled mean group (PMG) estimator developed by Pesaran et al. (1999). According to Pesaran et al. (1999), the PMG estimator takes the cointegration form of the panel ARDL model as shown in Eq. (7).

7 $$\Delta \mathrm{ln}{y}_{i,t}=\sum_{j=i}^{p-1}{\alpha }_{j}^{i}\Delta \mathrm{ln}{y}_{i,t-j}+\sum_{j=0}^{q-1}{\delta }_{j}^{i}\Delta {X}_{i,t-j}+ \varphi \left[\mathrm{ln}{Y}_{i,t-j}-\left\{{\beta }_{0}^{i}+{\beta }_{1}^{i}{X}_{i,t-j}\right\}\right]+{\mu }_{it}$$

where $\mathrm{ln}{y}_{i,t}$ represents the logarithm of the ecological footprint; *X* is a set of independent variables that include the private financial system and the interaction term; $\alpha$ and $\delta$ are short-term dynamic coefficients of the lagged dependent and independent variables, respectively; $\beta$ represents the long-term coefficients; $\varphi$ represents the coefficient of the adjustment speed to the long-term equilibrium; and $i$ and $t$ are the sub-indices that represent country and time, respectively. The full term in square brackets represents long-term regression, which includes the long-term coefficients of vectors *X*, resulting in Eq. (8).

8 $$\mathrm{ln}{y}_{i,t}={\beta }_{0}^{i}+{\beta }_{1}^{i}{X}_{i,t-j}+{\varepsilon }_{it}\mathrm{ where }{\varepsilon }_{i,t}\sim I\left(0\right)$$

#### Causality tests

Finally, to determine the existence and causal direction of the study variables, the panel causality test proposed by Dumitrescu and Hurlin (2012) is used, which is an approach to the Granger-type panel causality test (1988). In addition, this model applied the *bootstrap*, which allows to consider the cross-sectional dependence problem that occurs in cross sections. The model proposed by Dumitrescu and Hurlin (2012) is found in Eq. (9).

9 $$\mathrm{log}({EF}_{i,t}) = ({\alpha }_{i}+ \sum_{k=1}^{K}{\gamma }_{i}^{k}\mathrm{log}\left({EF}_{i,t-k}\right)+\sum_{k=1}^{K}{\beta }_{i}^{k}{x}_{i,t-k}+{\varepsilon }_{i,t}$$

where ${x}_{i,t-k}$ represents the independent variables used in this study. It is also assumed that ${\beta }_{i}={{\beta }_{i}}^{(1)}\dots {{\beta }_{i}}^{\left(k\right)}$ and ${\alpha }_{i}$ are fixed in time. On the other hand, ${\gamma }_{i}^{k}$ and ${\beta }_{i}^{k}$ represent the autoregressive parameter and the regression coefficient respectively, which vary between cross sections.

## Results and discussion

This section shows the results obtained from estimating the equations shown in the methodology for 100 countries. To achieve the objective of this research, some tests must be carried out in order to get to know the particularities of the data. This analysis will begin with the cross-sectional dependence test (CD), for which two tests, Pesaran (2004) and Pesaran (2015), will be used. Table 4 shows the results of these cross-sectional dependence tests, where the null hypothesis that states that there is no cross-sectional dependence (CD) between the study variables is rejected, given that the probability of the two tests used is less than 1%. This means that there are dependencies between countries, that is, if an alteration occurs in one country, it can be extended to the other countries involved in the study. In real terms, this scenario is due to the different economic, political, diplomatic and commercial relations, among others, that exist between the countries under study, which would lead to an interdependence between them. Therefore, there are interactions between countries, which means that the variation of some aspect in one country can be reflected in the behaviour of another country. It is also necessary to apply second-generation unit root tests to control the CD problem and analyse the seasonality of the variables considered in the study.

**Table 4:** Results of cross-sectional dependence (CD) tests

Variables	Pesaran (2004)		Pesaran (2015)	
	CD test	*P*-value	CD test	*P*-value
Log (ecological footprint per capita)	24.45***	0.000	242.50***	0.000
Log (private financial system)	111.94***	0.000	435.66***	0.000
Foreign direct investment	105.64***	0.000	262.44***	0.000
Log (urbanization)	266.54***	0.000	444.33***	0.000
Log (economic growth)	206.72***	0.000	444.67***	0.000

^***^ represents the significance level at 1%. ** represents the significance level at 5%. * represents the significance level at 10%

Table 5 shows the results of the second-generation unit root tests. In this regard, the cross-sectional augmented Dickey-Fuller test (CADF) and the cross-sectional augmented IPS test (CIPS) developed by Pesaran (2003) and Pesaran (2007), respectively, are used. These tests performed indicate that not all variables are stationary at level; in other words, the null hypothesis cannot be rejected at level. However, when considering second differences, all the variables become stationary, at a significance level of 1%, and are integrated in order two (II(2)).

**Table 5:** Results of the unit root tests of the second-generation panel

Income level	Variable	Cross-sectional augmented Dickey-Fuller (CADF)				Cross-sectional augmented IPS (CIPS)				Order	
		Level		Second difference		Level		Second difference			
		Intercept	Intercept and trend	Intercept	Intercept and trend	Intercept	Intercept and trend	Intercept	Intercept and trend		
100 countries	$\mathrm{Log}({EF}_{\mathrm{pc}})$	− 1.982**	− 1.917	− 5.242***	− 6.113***	− 2.468***	− 2.608**	− 5.209***	− 6.338***		II(2)
	$\mathrm{Log}(PFS)$	− 1.960**	− 2.542***	− 4.828***	− 6.168***	− 1.861	− 2.434	− 4.819***	− 6.390***		II(2)
	$FDI$	− 2.486***	− 2.593**	− 5.550***	− 6.144***	− 3.787***	− 4.019***	− 5.576***	− 6.366***		II(2)
	$\mathrm{Log}(URB)$	− 2.327***	− 2.903***	− 2.925***	− 4.743***	− 2.756***	− 3.407***	− 3.151***	− 4.982***		II(2)
	$\mathrm{Log}({GDP}_{\mathrm{pc}})$	− 2.033***	− 2.269	− 4.544***	− 6.154***	− 2.129**	− 2.477	− 4.519***	− 6.345***		II(2)
HIC	$\mathrm{Log}({EF}_{\mathrm{pc}})$	− 1.863	− 1.800	− 5.112***	− 6.064***	− 2.308***	− 2.485	− 5.050***	− 6.311***		II(2
	$\mathrm{Log}(PFS)$	− 2.171***	− 2.178	− 4.659***	− 6.154***	− 2.051*	− 2.121	− 4.664***	− 6.364***		II(2)
	$FDI$	− 2.368***	− 2.252	− 5.379***	− 6.136***	− 3.525***	− 3.732***	− 5.401***	− 6.362***		II(2)
	$\mathrm{Log}(URB)$	− 2.439***	− 1.236	− 3.180***	− 4.934***	− 2.652***	− 2.500	− 3.314***	− 5.058***		II(2)
	$\mathrm{Log}({GDP}_{\mathrm{pc}})$	− 2.345***	− 2.550*	− 4.211***	− 6.022***	− 2.336***	− 2.577*	− 4.206***	− 6.184***		II(2)
UMIC	$\mathrm{Log}({EF}_{\mathrm{pc}})$	− 2.173***	− 2.386	− 5.306***	− 6.190***	− 2.950***	− 3.040***	− 5.272***	− 6.420***		II(2)
	$\mathrm{Log}(PFS)$	− 1.836	− 2.110	− 4.712***	− 6.178***	− 1.692	− 2.051	− 4.677***	− 6.396***		II(2)
	*FDI*	− 2.377***	− 2.538	− 5.510***	− 6.190***	− 3.399***	− 3.560***	− 5.493***	− 6.419***		II(2)
	$\mathrm{Log}(URB)$	− 2.016*	− 2.435	− 3.055***	− 5.007***	− 2.515***	− 3.067***	− 3.275***	− 5.143***		II(2)
	$\mathrm{Log}({GDP}_{\mathrm{pc}})$	− 2.281***	− 2.554	− 4.532***	− 6.142***	− 2.322***	− 2.583*	− 4.451***	− 6.327***		II(2)
LMIC	$\mathrm{Log}({EF}_{\mathrm{pc}})$	− 2.105**	− 2.706**	− 5.323***	− 6.190***	− 2.070	− 3.085***	− 5.314***	− 6.411***		II(2)
	$\mathrm{Log}(PFS)$	− 1.842	− 2.524	− 5.273***	− 6.166***	− 1.918	− 2.716**	− 5.271***	− 6.376***		II(2)
	$FDI$	− 2.354***	− 2.372	− 5.660***	− 6.190***	− 3.963***	− 4.121***	− 5.671***	− 6.420***		II(2)
	$\mathrm{Log}(URB)$	− 2.099**	− 2.476	− 2.880***	− 4.887***	− 2.263**	− 2.706**	− 3.055***	− 5.002***		II(2)
	$\mathrm{Log}({GDP}_{\mathrm{pc}})$	− 1.702	− 2.283	− 4.532***	− 6.173***	− 1.664	− 2.189	− 4.472***	− 6.380***		II(2)
LIC	$\mathrm{Log}({EF}_{\mathrm{pc}})$	− 1.633	− 1.962	− 5.528***	− 6.190***	− 2.576***	− 2.937***	− 5.542***	− 6.420***		II(2)
	$\mathrm{Log}(PFS)$	− 2.318***	− 3.121***	− 4.737***	− 6.190***	− 2.124	− 2.734*	− 4.668***	− 6.420***		II(2)
	$FDI$	− 2.776***	− 2.924***	− 5.428***	− 6.136***	− 3.782***	− 3.833***	− 5.466***	− 6.323***		II(2)
	$\mathrm{Log}(URB)$	− 1.168	− 2.404	− 2.986***	− 4.011***	− 1.363	− 2.818**	− 3.333***	− 4.170***		II(2)
	$\mathrm{Log}({\mathrm{GDP}}_{\mathrm{pc}})$	− 1.455	− 2.916***	− 5.035***	− 6.190***	− 1.875	− 3.331***	− 5.023***	− 6.381***		II(2)

^***^ represents the significance level at 1%. ** represents the significance level at 5%. * represents the significance level at 10%

In order to perform the analysis of cointegration between the variables under study, we first performed the slope homogeneity test (Pesaran and Yamagata 2008). The results of this homogeneity test ($\widetilde{\Delta }$ 71.436, *P*-value = 0.000; $\widetilde{\Delta }$ adj 77.483, *P*-value = 0.000) indicate that the null hypothesis stating that there is homogeneity in the slope is rejected, and it is observed that the slopes of the variables vary according to the country. The data obtained indicate that a cointegration test that addresses the problems of cross-sectional dependence (CD) and heterogeneity of the data should be applied, the panel cointegration test of Westerlund (2007).

As can be seen in the Westerlund test (2007), four tests of group and panel cointegration are presented (Table 6). According to the calculated values, at a significance level of 1%, the null hypothesis is rejected, and it is confirmed that at least one unit of the cross section is cointegrated and that the panel as a whole is cointegrated, that is, that there is a joint movement between the variables in the long term. This result confirms that a change in the private financial system and control variables such as foreign direct investment, urbanization and economic growth will lead to changes in the ecological footprint of the 100 study countries in the long term. In this regard, these results are consistent with those obtained by Usman et al. (2020) and Godil et al. (2020), who observed in their research that there is co-movement between the series over time. Similarly, the long-term cointegration results coincide with those found by Sharma et al. (2021c) and Sharma et al. (2020), when examining long-term environmental degradation in Asian economies.

**Table 6:** Results of the panel-data cointegration test (Westerlund test)

Income level	Statistic	Value	*Z*-value	*P*-value	*P*-robust value
100 countries	*G*_*t*_	− 6.523	− 44.189	0.000	0.000
	*G*_*a*_	− 57.870	− 67.483	0.000	0.000
	*P*_*t*_	− 75.743	− 47.024	0.000	0.000
	*P*_*a*_	− 68.333	− 85.102	0.000	0.000
HIC	*G*_*t*_	− 6.571	− 25.262	0.000	0.000
	*G*_*a*_	− 66.030	− 44.665	0.000	0.000
	*P*_*t*_	− 43.330	− 26.982	0.000	0.000
	*P*_*a*_	− 69.488	− 49.035	0.000	0.000
UMIC	*G*_*t*_	− 6.682	− 23.317	0.000	0.000
	*G*_*a*_	− 53.547	− 31.310	0.000	0.000
	*P*_*t*_	− 34.214	− 20.499	0.000	0.000
	*P*_*a*_	− 53.368	− 32.957	0.000	0.000
LMIC	*G*_*t*_	− 6.322	− 21.942	0.000	0.000
	*G*_*a*_	− 55.305	− 33.191	0.000	0.000
	*P*_*t*_	− 32.626	− 19.121	0.000	0.000
	*P*_*a*_	− 56.848	36.058	0.000	0.000
LIC	*G*_*t*_	− 6.509	− 17.059	0.000	0.000
	*G*_*a*_	− 52.574	− 23.252	0.000	0.000
	*P*_*t*_	− 24.579	− 14.458	0.000	0.000
	*P*_*a*_	− 54.178	− 25.461	0.000	0.000

Finally, panel ARDL models are applied in order to analyse the relationship in the short and long terms of the variables. These allow us to examine the coefficients of the variables in the long term and examine their economic implications (Zhang et al. 2022; Sharma and Kautish 2020). Dynamic mean group (MG) and pooled mean group (PMG) estimators are used. To choose between one estimator or another, the Hausman test was applied. The results of the Hausman test showed that the Prob > chi^2^ is greater than 0.05 for the 100 countries, HIC, LMIC and LIC. Thus, the PMG estimator is the one used in these cases. However, for UMIC, the Prob > chi^2^ is equal to 0.012, (less than 0.05), so the most appropriate estimator for this case will be the mean group (MG) estimator. The MG and PMG estimator show two columns of equations. The first column shows the normalised cointegration equation and the second one, the short-term dynamics. Table 7 reports the results of the panel ARDL estimates, showing the long- and short-term relationship of the dynamic coefficients. The first column provides the long-term relationship, showing the variables that are significant in the countries according to income level.

**Table 7:** Panel ARDL estimation results

Variables	100 countries		HIC		UMIC		LMIC		LIC	
	PMG		PMG		MG		PMG		PMG	
	Long-term	Short-term	Long-term	Short-term	Long-term	Short-term	Long-term	Short-term	Long-term	Short-term
Error correction (ECM)		− 0.423***		− 0.499***		− 0.989***		− 0.431***		− 0.539***
		(0.000)		(0.000)		(0.000)		(0.000)		(0.000)
$\mathrm{Log}(PFS)$	0.004	− 0.008	− 0.151***	− 0.016	0.081***	0.006	0.035***	0.002	0.043***	0.004
	(0.626)	(0.814)	(0.000)	(0.881)	(0.007)	(0.819)	(0.000)	(0.887)	(0.000)	(0.789)
*FDI*	0.002***	0.002	0.001	0.008	0.010*	− 0.003	0.005***	− 0.001	0.002	0.002
	(0.002)	(0.441)	(0.504)	(0.226)	(0.077)	(0.186)	(0.000)	(0.334)	(0.108)	(0.391)
$\mathrm{Log}(URB)$	− 0.118***	− 11.482	− 0.614***	− 32.548	0.361*	− 4.833	0.162***	− 3.973**	− 0.237***	0.209
	(0.000)	(0.480)	(0.000)	(0.507)	(0.076)	(0.396)	(0.000)	(0.039)	(0.000)	(0.918)
$\mathrm{Log}({\mathrm{GDP}}_{\mathrm{pc}})$	0.509***	0.605***	0.591***	0.733**	0.267***	0.469***	0.456***	0.338***	0.112***	0.376***
	(0.000)	(0.000)	(0.000)	(0.025)	(0.001)	(0.000)	(0.000)	(0.003)	(0.000)	(0.005)
Constant		− 1.314***		− 0.672***		− 3.611***		− 1.658***		0.038
		(0.000)		(0.000)		(0.002)		(0.000)		(0.490)
Remarks	3800	3800	1216	1216	936	936	936	936	570	570
Groups/countries	100	100	32	32	26	26	26	26	15	15
Hausman test	0.195		0.060		0.001		0.151		0.676	

^***^ represents the significance level at 1%. ** represents the significance level at 5%. * represents the significance level at 10%

In the second column, the short-term dynamism is shown, where the error correction term (ECM) is found, which is the adjustment speed to reach long-term equilibrium and is of main interest, since it must be proven that this term is negative and statistically significant. According to the level of income, it can be seen that the ECM is negative and statistically significant at the global, HIC, UMIC, LIMIC and LIC levels. With the results found, it can be concluded that there is convergence to equilibrium in the long term in the variables under study.

The results estimated at the level of the 100 countries and according to income level show that there is a long-term relationship. With regard to the private financial system, which is measured by domestic credits to the private sector, and its relationship with the ecological footprint, it can be seen that at the level of the 100 countries, both in the short and long terms, it is not significant. In the case of HIC, the private financial system has a significant negative impact on the long-term ecological footprint. A 1% increase in domestic credits to the private sector will reduce the ecological footprint by 0.151%. This result indicates that the growth of the private financial system plays a key role in mitigating the environmental degradation of HIC. These results are consistent with those obtained by Dogan et al. (2019) and Usman and Hammar (2021), who showed that the relationship between the private financial system and the ecological footprint is adverse. Likewise, Villanthenkodath and Arakkal (2020) stated that this negative relationship is due to the fact that people demand a quality environment, having greater access to credit. In addition, an increase in financial resources will allow competitive companies to focus on acquiring techniques and technology that contribute to improving environmental quality (Sharma et al. 2021c).

On the other hand, with regard to UMIC, LMIC and LIC, the financial system is statistically significant in the long term and its effect is positive on the ecological footprint. This suggests that a 1% increase in domestic *PFS* (credits to the private sector) will increase the ecological footprint by 0.081, 0.035 and 0.043% in UMIC, LMIC and LIC, respectively. This finding implies that the growth of the private financial system will be different depending on the income that countries have. In the case of UMIC, LMIC and LIC, this leads to an increase in the ecological footprint, due to the fact that entrepreneurs invest in activities that generate a higher profit, without taking the environmental impact into account. Similar results were shown in the study developed by Nathaniel and Adeleye (2021) and Ahmed et al. (2021), who state that an increase in credits by the private financial system causes a greater demand for natural resources such as land and water, among others.

Foreign direct investment (*FDI*) is significant only at the level of the 100 countries and in LMIC. However, its impact is insignificant, given that a 1% increase in *FDI* will increase the ecological footprint by 0.002% and 0.005%, respectively. This effect is due to the fact that *FDI* is minimal in some countries, due to the lack of economic and trade policies that favour its effect. Results were corroborated by Mahmood et al. (2020), who pointed out that the effect of *FDI* on environmental quality is statistically insignificant. Moreover, its positive effect coincides with the findings by Opoku and Boachie (2020) and Murshed et al. (2021), who argue that developing countries maintain weak environmental regulations, attracting highly polluting *FDI*. In contrast, studies such as those by Zafar et al. (2019), Eluwole et al. (2020) and Xie et al. (2020) argue that there is a significant, but negative relationship between *FDI* and the ecological footprint, as they attract *FDI* that contributes with efficient and sustainable techniques and technology, which reduces the ecological footprint.

On the other hand, regarding the relationship between urbanization and the ecological footprint, the results are statistically significant at the level of the 100 countries, HIC, LMIC and LIC in the long term, but their effect differs according to the income level of the countries. At the level of the 100 countries, HIC and LIC, their effect is negative. These results are consistent with Danish and Wang (2019), who argue that the negative effect of urbanization on the ecological footprint is due to the fact that it generates positive externalities in the environment. Similarly, Dogan et al. (2019) established that the impact of urbanization is negative on the long-term ecological footprint for Nigeria, demonstrating the difference in the urban planning techniques applied,

On the other hand, a particular situation arises in LMIC, where urbanization is significant in the short and long terms, but its impact differs, being positive in the long term and negative in the short term. Specifically, a 1% increase in urban population will decrease the ecological footprint by 3.97% in the short term and increase the ecological footprint by 0.16% in the long term. This result indicates that the measures and projects adopted by LMIC for the construction of infrastructure mitigate the environmental impact generated by the urban population in the short term. However, in the long term, it will increase the ecological footprint. Similar findings were made by Ridzuan et al. (2020), who stated that urbanization decreases environmental degradation in the short term by emitting less CO_2_, as the urban population begins to develop and implement health infrastructures, as well as improve transport systems, among others, which reduces pollution. However, in the long term, its effect will be the opposite. In contrast, Ahmed et al. (2020) and Langnel and Amegavi (2020) argued that the effect of urbanization on the ecological footprint is positive in the short and long term. According to Ahmed et al. (2020), Ulucak et al. (2020) and Langnel and Amegavi (2020), it is due to the fact that urbanization impacts on economic and social activities, which demand higher energy consumption in households and production sectors. In addition, they do not have planned urban systems infringing on greater environmental pressures (Ulucak et al. 2020).

Finally, the estimate of economic growth is positive and has a considerable effect on the ecological footprint in both the short and long terms. This suggests that a higher gross domestic product per capita will increase environmental degradation, at the level of the 100 countries, in HIC, UMIC, LMIC and LIC, both in the long and short terms. This finding mainly indicates that the economic growth of a country increases consumption, spending, investment and marketing activities, which demand a greater amount of natural resources, energy use, as well as generating high amounts of waste that cause the ecological footprint to continue to increase, as they are harmful processes in which environmental quality is affected (Shujah-ur-Rahman et al. 2019d). Similar long-term findings were obtained by Nathaniel and Khan (2020), Ulucak et al. (2019) and Uddin et al. (2019), who corroborated that in the long term, an increase in gross domestic product will increase the ecological footprint. However, environmental policies need to be established progressively in order to reduce the environmental impact that will be incurred.

Table 8 shows Dumitrescu-Hurlin panel causality test (Dumitrescu and Hurlin 2012). The results show the existence of a causal relationship between environmental degradation, relationship is bidirectional (measured by the ecological footprint) and the private financial system (measured by domestic credits to the private sector) at the global, HIC, UMIC and LMIC levels. In this regard, it is established that changes produced in private credits will cause variations in the ecological footprint and vice versa. These results are similar to those obtained by Usman et al. (2020), Usman and Hammar (2021) and Aluko and Obalade (2020), who found a bidirectional causal relationship between the ecological footprint and the private financial system, indicating the importance of a stable and efficient financial structure in which environmental regulations are integrated. In the same vein, Shujah-ur-Rahman et al. (2019c) state that there is a bidirectional relationship between the private financial system and the ecological footprint, highlighting the need for strict environmental regulations that encourage green investments.

**Table 8:** Dumitrescu-Hurlin panel causality test

Null hypothesis		100 Countries	HIC	UMIC	LMIC	LIC
$\mathrm{Ln}{EF}_{pc}$ no cause $\mathrm{Ln}SFP$	*Z*-bar	5.3150***	2.8671**	3.7008***	1.7446	2.3227**
	*P*-value	0.0000	0.0500	0.0000	0.1400	0.0400
$\mathrm{Ln}PFS$ no cause $\mathrm{Ln}{EF}_{pc}$	*Z*-bar	5.0248***	4.1550***	3.4425***	1.7209	0.0644
	*P*-value	0.0000	0.0057	0.0200	0.1500	0.9800
$\mathrm{Ln}{EF}_{pc}$ no cause *F* $DI$	*Z*-bar	2.2777**	0.4828	2.0248	0.9296	1.2629
	*P*-value	0.0400	0.6100	0.1400	0.5000	0.1900
$F$ *DI* no cause $\mathrm{Ln}{EF}_{pc}$	*Z*-bar	0.7921	2.6225**	0.3914	− 0.0060	1.2618
	*P*-value	0.6000	0.0300	0.6800	1.0000	0.2300
$\mathrm{Ln}{EF}_{pc}$ no cause $\mathrm{Ln}URB$	*Z*-bar	0.4734	− 0.0813	− 1.9482*	1.3725	2.0646*
	*P*-value	0.6400	0.9200	0.0800	0.1900	0.0600
$\mathrm{Ln}URB$ no cause $\mathrm{Ln}{EF}_{pc}$	*Z*-bar	− 0.9404	− 0.8715	− 1.1974	1.3148	− 1.3427
	*P*-value	0.6100	0.4900	0.3600	0.2100	0.2700
$\mathrm{Ln}{EF}_{pc}$ no cause $\mathrm{Ln}{GDP}_{pc}$	*Z*-bar	2.2937**	3.0404**	− 0.0368	0.5610	0.7772
	*P*-value	0.0200	0.0200	0.9900	0.6500	0.4700
$\mathrm{Ln}{GDP}_{pc}$ no cause $\mathrm{Ln}{EF}_{pc}$	*Z*-bar	2.4748*	2.3294*	− 0.0807	0.6040	2.2835*
	*P*-value	0.0900	0.0600	0.9400	0.4900	0.0600

^****^ represents the significance level at 1%. ** represents the significance level at 5%. * represents the significance level at 10%

On the other hand, for LIC, it is established that there is a unidirectional causal relationship of the ecological footprint to the financial system, which means that variations in the ecological footprint will cause changes in the private financial system. These results are consistent with studies by Destek and Sarkodie (2019), showing a similar relationship. However, it contradicts the results obtained by Dogan et al. (2019), who state that a change in the financial system will cause changes in the ecological footprint. On the other hand, with regard to the control variables, a unidirectional causal relationship of *FDI* at the global level and from the ecological footprint to *FDI* in HIC was observed. Nevertheless, there are studies that contradict these results, such as the one carried out by Sabir et al. (2020) and Tiba and Belaid (2020), who claim that the causal relationship between the variables is bidirectional, since capital flows can lead to changes in the environmental footprint, and similarly, environmental policies will restrict and encourage different *FDI* flows into economies. Likewise, Zafar et al. (2019) confirm a bidirectional relationship between the variables for the USA.

In terms of GDP per capita and the ecological footprint, a unidirectional causal relationship of economic growth to the ecological footprint was observed at the level of the 100 countries and in HIC. In other words, variations in the gross domestic product cause changes in the ecological footprint. This result indicates that economic growth implies a greater development of productive activities, generating pollutants and waste that increase the ecological footprint. These results are similar to those obtained by Wang et al. (2020a, b) and Danish Ulucak and Khan (2020), showing a unidirectional causal relationship that goes from economic growth to ecological footprint, at a significance level of 5%, stating that economic growth increases the demand for natural resources, which causes an increase in the ecological footprint. However, they contrast with Sharif et al. (2020) and Ahmad et al. (2020), who found bidirectional causality between the variables.

## Conclusions and policy implications

Environmental degradation related to the private financial system, foreign direct investment, urbanization and gross domestic product have an equilibrium relationship in the long term, at the global level and according to the income levels of countries. This implies that the variables are synchronised over time. Similarly, when applying the mean group (MG) and pooled mean group (PMG) estimators, the effect of the variables on environmental degradation was observed, taking the income level of the countries into account. For HIC, the private financial system plays a fundamental role in reducing environmental degradation in the long term. This impact is attributed to the fact that the financial sector is better structured in HIC and financial resources are allocated to projects that prioritise environmental sustainability and efficiency. With regard to UMIC, LMIC and LIC, the private financial system is responsible for increasing the long-term ecological footprint because financial intermediaries focus their resources on productive sectors that increase environmental damage.

With regard to the control variables, at the panel, HIC and LIC level, urbanization mitigates long-term environmental degradation, since the increase in urban population maintains a growing trend over time, having multiple challenges, as well as an opportunity to minimise environmental damage. In this regard, urbanization represents a solution to reduce the ecological footprint, as innovative and environmentally efficient urban systems are required and implemented. On the other hand, in LMIC, a particular situation is shown, urbanization generates a decrease in environmental degradation in the short term and an increase in environmental degradation in the long term, due to the fact that the measures and projects for the construction of infrastructures that LMIC have adopted, mitigate the environmental impact generated by the urban population in the short term. Regarding economic growth, it positively affects the ecological footprint, both in the short and long terms, at the panel, HIC, UMIC, LMIC and LIC levels, since economic growth increases environmental problems, given the demand for a greater amount of resources by countries to sustain their model of development and economic growth. This means that it is an associated factor in environmental degradation. In addition, it reflects the need to substitute production and consumption patterns for others that are efficient and sustainable over time.

Furthermore, the results of the Dumitrescu and Hurlin (2012) test verify the existence of causality between pairs of variables. At the global level, in HIC and UMIC, the existence of bidirectional causality between the ecological footprint and the private financial system is determined. In other words, variations in the private financial system cause changes in the ecological footprint and vice versa. In contrast, in LIC, there is a unidirectional causality from the ecological footprint to environmental degradation. With regard to the control variables, foreign direct investment (*FDI*) has a unidirectional causal relationship from the ecological footprint to *FDI* at the global level and from *FDI* to the ecological footprint in HIC. In terms of economic growth, there is unidirectional causality from the ecological footprint to economic growth at the global level and in HIC, which means that policies affecting economic growth will impact on the ecological footprint.

Consequently, in line with the above results, it is concluded that the private financial system, urbanization and GDP per capita have significant positive and negative effects on the ecological footprint, according to the income level of the countries in the long term. In this sense, policy makers must take the particular behaviour of each of the variables into account when formulating policies. In the case of HIC, it is crucial to reduce environmental degradation in the long term, so governments are recommended to strengthen and promote the development of financial institutions and markets through reforms that grant regulatory flexibility in their operations, allowing them to expand and encourage private credits for environmentally sustainable projects.

With regard to upper middle-income countries (UMIC), lower middle-income countries (LMIC) and low-income countries (LIC), it is necessary for the State to implement strict environmental regulations that condition the private financial sector to channel its resources to projects that mitigate pollution such as organic agriculture, waste management and clean energy generation, among others, and, in turn, restrict them to heavily polluting companies. In addition, there should be joint work between private and public banks to promote environmental credit lines, offering benefits such as lower interest rates compared to conventional loans, with the aim of boosting investment in sustainable goods and activities.

Secondly, in HIC, UMIC and LIC, it is important to encourage a planned urbanization process that enables resources to be managed in a sustainable way, which requires coordination between national and municipal governments to redesign and develop environmentally friendly infrastructures using the least polluting materials, as well as increasing green urban areas, which contribute to carbon sequestration. Governments should also encourage the development of enterprises that offer sustainable goods and services through financial subsidies and by linking them to public projects.

Finally, the governments of the countries under study are recommended to implement circular economy processes that promote the efficient use of resources, promoting a change in the traditional production and consumption model by reusing natural resources and producing the least amount of waste. Moreover, as their income increases, a larger budget should be allocated to innovative projects to reduce pollution and pressure on environmental resources, making available to households and companies a variety of environmentally friendly alternatives based on the use of clean technologies and renewable energy-based electricity. In addition, the financial sector is required to create new financing strategies for projects, which are directed towards activities that take into account environmental conservation, considering the particularities of the countries according to their income level. On the other hand, the urbanization process must be attached to environmental sustainability, considering improving the way cities grow, aiming to create sustainable cities and avoid environmental degradation.

Some limitations of the study are related to the availability of information for all the countries examined, especially of variables that capture the updating of production processes, measured by technological progress or technological innovation. Consequently, this limitation leads to new research edges. Therefore, an extension of this study is to examine how technology affects environmental degradation, considering countries worldwide.

## Data Availability

The datasets used and/or analysed during the current study are available from the corresponding author on reasonable request.
